# Cross-Cultural Adaptation and Psychometric Testing of the Italian Barriers to Nursing Research Participation (I-BNPRQ)

**DOI:** 10.3390/healthcare14121793

**Published:** 2026-06-22

**Authors:** Mattia Bozzetti, Alessio Lo Cascio, Michela Colalelli, Piergiorgio Martella, Roberta Pendoni, Michela Piredda, Joseph Hagan, Monica Guberti, Daniele Napolitano

**Affiliations:** 1 Direction of Health Professions, ASST Cremona, Viale Concordia 1, 26100 Cremona, Italy; mattia.bozzetti@asst-cremona.it (M.B.); roberta.pendoni@asst-cremona.it (R.P.); 2 Direction of Health Professions, La Maddalena Cancer Center, via san Lorenzo 312 D, 90146 Palermo, Italy; locascio.alessio@lamaddalenanet.it; 3 Hematology, Fondazione Policlinico Gemelli IRCCS, Largo Agostino Gemelli 8, 00136 Rome, Italy; michela.colalelli@policlinicogemelli.it; 4 Neuroscience Department, Fondazione Policlinico Gemelli IRCCS, Largo Agostino Gemelli 8, 00136 Rome, Italy; piergiorgio.martella@policlinicogemelli.it; 5 Research Unit Nursing Science, Department of Medicine and Surgery, Campus Bio-Medico di Roma University, via Alvaro del Portillo 21, 00128 Rome, Italy; 6 Department of Pediatrics, Section of Neonatology, Baylor College of Medicine, Houston, TX 77003, USA; jlhagan@bcm.edu; 7 Nursing and Allied Health Professions Directorate, IRCCS Istituto Ortopedico Rizzoli, Via Alessandro Codivilla, 40136 Bologna, Italy; monica.guberti@ior.it; 8 SITRA and Scientific Direction, Fondazione Policlinico Gemelli IRCCS, Largo Agostino Gemelli 8, 00136 Rome, Italy

**Keywords:** nursing research, psychometric validation, cultural adaptation, barriers to research participation, Italian nurses, exploratory structural equation modelling, evidence-based practice

## Abstract

**Highlights:**

**What are the main findings?**

- The Italian Barriers to Nurses’ Participation in Research Questionnaire (I-BNPRQ) was cross-culturally adapted for the Italian cultural and organisational healthcare context and showed preliminary evidence of structural validity, internal consistency, and temporal stability.
- The final Italian version retained a two-factor structure but required culturally and psychometrically justified item-level refinements, including the exclusion of items with weak contribution to the latent structure and the merging of two conceptually overlapping items.

**What are the implications of the main findings?**

- The questionnaire may support the identification of organisational and individual barriers to nurses’ research participation in the Italian context.
- Targeted institutional strategies, protected research time, and research education may help strengthen nurses’ engagement in research and, consequently, evidence-based care.

**Abstract:**

**Background/Objectives**: Nurses’ engagement in research is essential to strengthen evidence-based practice, knowledge translation, and quality of care. However, individual, organisational, and cultural barriers may limit nurses’ participation in research activities. This study aimed to cross-culturally adapt and psychometrically test the Italian version of the Barriers to Nurses’ Participation in Research Questionnaire within the Italian cultural and healthcare organisational context, and to explore perceived obstacles to research engagement among nurses in Italy. **Methods**: A cross-sectional methodological study was conducted. The instrument was translated, back-translated, reviewed by the original instrument developer and an expert panel, and evaluated for content validity by 12 clinical research professionals. Data were collected online between September and October 2024 from 196 nurses working across Italian healthcare settings, including hospitals, university hospitals, IRCCS, primary care, and private hospitals. Exploratory Structural Equation Modelling was used to examine the factor structure. **Results**: A total of 196 nurses were enrolled in the study. A two-factor structure was identified, comprising *Research Resources* and *Personal Relevance of Research*, which explained 35.37% and 25.14% of the variance, respectively. Both factors demonstrated good reliability. The most prominent barrier was the lack of incentive or reward for nurses to engage in research, whereas the least relevant barrier was the perception that research was not interesting or valuable. Greater barriers were reported by younger nurses, those with fewer years of experience, and those without specific research training. Lack of time to conduct research emerged as a pervasive obstacle across the sample. **Conclusions**: The Italian version of the *Barriers to Nurses’ Participation in Research Questionnaire* provides preliminary evidence of validity and reliability for assessing perceived barriers to research participation among Italian nurses. Owing to the structural modifications introduced during adaptation, the instrument should be interpreted as a culturally adapted and modified Italian version rather than as a direct replication of the original structure. Its use may support organisational diagnosis, research mentorship, training planning, and future research-capacity-building initiatives, although further validation in larger and more heterogeneous samples is warranted.

## 1. Introduction

Nurses’ participation in research is essential for advancing clinical knowledge, strengthening evidence-based nursing, supporting knowledge translation, and improving the quality and safety of patient care. Beyond applying evidence in routine practice, nurses contribute to the generation, appraisal, implementation, and translation of research findings into clinical decision-making and service redesign [1,2,3,4]. Despite this recognised importance, engagement in research remains uneven across settings and countries, and many nurses continue to experience substantial barriers to active research participation [5,6].

Research engagement in nursing should be understood as a multidimensional process that includes awareness of research evidence, participation in research activities, use of research findings in clinical decision-making, and contribution to knowledge translation within healthcare organisations. In this perspective, research participation is not limited to the production of scientific evidence, but also includes the capacity to interpret, implement, and sustain evidence-informed practices in clinical settings. These dimensions are closely aligned with evidence-based nursing and implementation science, both of which emphasise the importance of organisational readiness, professional competencies, leadership support, and contextual factors in translating evidence into routine care.

Previous literature has identified multiple barriers that may limit nurses’ involvement in research. These barriers can be conceptualised at individual, organisational, and cultural levels. Individual barriers include limited research knowledge, low confidence, lack of methodological training, and uncertainty about the personal relevance of research. Organisational barriers include insufficient institutional support, limited access to mentorship and infrastructure, lack of protected time, scarce professional development opportunities, and weak recognition of research-related roles within clinical organisations [7,8,9,10]. These barriers are not only individual but also organisational and cultural, reflecting the extent to which research is integrated into nursing roles, supported by leadership, and valued within healthcare institutions [11,12]. Conversely, facilitators of research participation include supportive leadership, access to infrastructure and networks, tailored educational opportunities, and organisational strategies that align research activities with nurses’ professional interests and career development [13,14].

In Italy, the integration of nurses into research activities appears to reflect many of these international challenges. Although nursing research has progressively gained visibility, its development is still often driven by a limited number of highly motivated professionals, and research-related roles remain inconsistently recognised across institutions [15,16,17]. In particular, unclear boundaries between clinical, research-support, and research-leadership roles may hinder nurses’ full participation in research activities and contribute to uncertainty regarding expectations, responsibilities, and opportunities [18,19,20]. These contextual features make it especially important to identify how Italian nurses perceive barriers to research participation and to do so using a psychometrically sound instrument [21,22].

In the Italian context, these issues are particularly relevant because nurses’ involvement in research remains heterogeneous across healthcare organisations. While some university hospitals and Scientific Institutes for Research, Hospitalisation and Healthcare have progressively developed research-support roles and clinical research infrastructures, many clinical settings still lack formalised pathways for nurses’ research engagement, protected research time, or structured recognition of research-related competencies. This organisational variability may affect nurses’ perception of research as either an integral component of professional practice or an activity external to routine clinical responsibilities. Among the available instruments, the BNPRQ was selected because it specifically assesses perceived barriers to nurses’ participation in research rather than measuring only research competencies, evidence-based practice attitudes, or research utilisation. This distinction is relevant because the purpose of the present study was not to evaluate nurses’ ability to conduct research, but to identify the perceived individual and organisational obstacles that may hinder their involvement in research activities. Moreover, the BNPRQ was developed from prior work on hospital-based nursing research barriers and includes dimensions directly related to research resources, personal relevance, and time availability. Therefore, it was considered particularly suitable for exploring research participation barriers within the Italian nursing context. To date, no validated Italian-language version of the BNPRQ is available, limiting the ability to systematically assess research barriers in the Italian nursing context and to compare findings across settings.

## 2. Materials and Methods

### 2.1. Design

This was a cross-sectional methodological study involving the translation, cross-cultural adaptation, and psychometric evaluation of the BNPRQ for use among Italian nurses. This study is reported in accordance with the Strengthening the Reporting of Observational Studies in Epidemiology (STROBE) checklist [23]. A convenience sampling strategy was adopted to recruit nurses from different Italian healthcare settings. This approach was considered appropriate for the exploratory validation phase of a culturally adapted instrument, although its implications for generalisability are acknowledged in the limitations section.

### 2.2. Measures

#### 2.2.1. Barriers to Nurses’ Participation in the Research Questionnaire

The original BNPRQ is a 15-item instrument developed and validated in a large academic paediatric hospital in the United States [24]. It was designed to assess perceived barriers to nurses’ participation in research and includes two subscales: Research Resources and Personal Relevance of Research, as well as one standalone item addressing time available for research, which has frequently been identified as a major barrier in the literature. Items are rated on a five-point Likert scale, with higher scores indicating greater perceived barriers. In the original study, internal consistency was acceptable (Research Resources α = 0.79, Personal Relevance of Research α = 0.74) [24]. The survey also included two single-item questions assessing satisfaction with research opportunities and personal involvement in research: “How satisfied are you with the opportunities for nurses to engage in research within your institution?” and “How satisfied are you with your own participation in research?”, both rated on a five-point scale from 1 (“not at all satisfied”) to 5 (“very satisfied”).

#### 2.2.2. Translation and Cross-Cultural Adaptation

The BNPRQ developers authorised its use [24]. The adaptation followed international guidelines [25]. Two independent forward translations from English into Italian were performed by translators proficient in English and Italian. One translator had knowledge of healthcare terminology and nursing research, while the other was not directly involved in the construct under investigation, in order to balance conceptual accuracy and natural language use. The project leader and translators compared the two forward translations with the original English version and produced a reconciled Italian version. Two independent back-translations into English were then performed by professional translators who were unaware of the original questionnaire and study aims. The back-translated versions were compared with the original BNPRQ and sent to the developers of the original instrument for review. This step was particularly important because one of the original developers was also involved as a co-author in the present study, ensuring that item-level decisions were discussed with direct knowledge of the original conceptual framework. A multidisciplinary expert panel, including translators, nurse researchers, clinical research nurses, psychologists, and healthcare professionals with experience in research methodology and questionnaire adaptation, reviewed all translated versions. The panel assessed semantic, idiomatic, experiential, and conceptual equivalence. Semantic equivalence concerned the literal meaning of each item; idiomatic equivalence concerned the naturalness of expressions in Italian; experiential equivalence concerned the relevance of each item to the Italian nursing context; and conceptual equivalence concerned the preservation of the construct measured by the original BNPRQ. Discrepancies were resolved through discussion until consensus was reached.

No formal cognitive debriefing or pilot testing with a separate sample of target users was conducted before the psychometric field testing. However, the multidisciplinary expert review and the involvement of the original BNPRQ developer supported the assessment of conceptual and cultural equivalence before administration. The absence of a formal cognitive debriefing phase is acknowledged as a limitation of the study.

#### 2.2.3. Content Validity

Twelve experts, including clinical research nurses, research managers, and nurse researchers, evaluated the content validity of the I-BNPRQ. They assessed each item’s relevance and the overall questionnaire using a 3-point scale (0 = “not essential”, 1 = “useful”, 2 = “essential”) [26]. Open-ended questions invited feedback on clarity, coherence, potential gaps, and suggestions for additions or changes. Substantial differences in expert opinions led to revisions to the items through repeated discussions until consensus was achieved. The Content Validity Ratio was calculated for each item according to Lawshe’s method. For the 12-member expert panel, all items reached acceptable CVR values, ranging from 0.667 to 1.000, and were therefore considered relevant for inclusion at the content validity stage. Items with lower CVR values were discussed by the expert panel to verify their cultural and conceptual relevance in the Italian context. Consensus was reached through iterative discussion, with particular attention to semantic clarity, conceptual equivalence, and applicability to Italian nursing practice. The complete CVR values are reported in Supplementary File S1.

#### 2.2.4. Data Collection

Participants were recruited using a convenience sampling strategy between September and October 2024. Eligible participants were registered nurses working in Italian healthcare settings, including public hospitals, private hospitals, university hospitals, Scientific Institutes for Research, Hospitalisation and Healthcare, and primary care services. Nurses were eligible if they were currently working in clinical, educational, managerial, or research-related roles and were able to complete the questionnaire in Italian. Nurses who did not provide informed consent or who submitted incomplete questionnaires were excluded from the analysis.

Recruitment was conducted through professional networks, continuing education activities, and contacts with clinical and research settings. The online questionnaire included the I-BNPRQ, two satisfaction items related to research opportunities and personal research participation, and socio-demographic and professional variables, including gender, education, geographic area, age, type of institution, clinical specialisation, job position, professional experience, prior research experience, research training, and perceived adequacy of university education for research.

The sample included nurses with different levels of exposure to research, including nurses involved in continuing education activities, nurses engaged in clinical research contexts, and nurses without formal research training working in clinical settings. This heterogeneity was considered useful for exploring perceived barriers across different professional profiles, although the non-probabilistic sampling strategy limited the possibility of performing robust stratified subgroup analyses.

### 2.3. Statistical Analyses

Descriptive statistics were used to summarise sample characteristics and item distributions. Continuous variables were reported as mean and standard deviation (SD), while categorical variables were summarised as frequencies and percentages.

Because the original BNPRQ was developed with a theoretically defined two-factor structure, this structure was first examined using Confirmatory Factor Analysis (CFA) [27,28]. However, given the possibility of item cross-loadings in a culturally adapted version of a multidimensional instrument, Exploratory Structural Equation Modelling (ESEM) was used as the primary approach to evaluate dimensionality [26,29,30]. ESEM combines the flexibility of exploratory factor analysis with the model-testing advantages of confirmatory approaches and is particularly useful when some degree of cross-loading is theoretically plausible [29,31]. Before model estimation, the suitability of the correlation matrix for factor analysis was assessed using Bartlett’s test of sphericity and the Kaiser–Meyer–Olkin (KMO) measure of sampling adequacy. Given the ordinal nature of the item responses and evidence of non-normality, models were estimated using a robust maximum likelihood estimator (MLR). MLR estimation was considered acceptable given the five-category response format and the exploratory ESEM framework. Within the ESEM framework, target rotation was applied to guide factor loadings according to the expected factor structure while allowing cross-loadings to be freely estimated. Model fit was evaluated using the chi-square statistic (χ^2^), Comparative Fit Index (CFI), Tucker–Lewis Index (TLI), Standardised Root Mean Square Residual (SRMR), and Root Mean Square Error of Approximation (RMSEA). Acceptable model fit was defined as CFI and TLI values of at least 0.90, SRMR values of 0.08 or lower, and RMSEA values of 0.08 or lower, with consideration of the 90% confidence interval (CI). The chi-square statistic was interpreted cautiously because of its sensitivity to sample size.

Item-level decisions were made through an integrated empirical and theoretical process rather than on statistical criteria alone. Items were examined when they showed low standardised loadings, limited contribution to the latent factor structure, substantial cross-loading, conceptual redundancy, or problematic interpretability across alternative specifications. Decisions regarding item retention, removal, and merging were discussed by the research team and interpreted in light of the original BNPRQ conceptual framework. Importantly, one of the developers of the original BNPRQ was involved as a co-author in the present study; therefore, refinements were considered not as arbitrary statistical modifications but as culturally informed adaptations aimed at preserving conceptual coherence while improving the interpretability of the Italian version.

To explore construct validity, associations between I-BNPRQ scores and continuous covariates were examined using Spearman’s rank correlation coefficient (ρs). Internal consistency was assessed using McDonald’s omega because omega provides a less restrictive estimate of reliability than Cronbach’s alpha when tau-equivalence cannot be assumed, as is often the case in multidimensional or structurally complex instruments. This choice was considered particularly appropriate given the ESEM framework and the presence of potentially heterogeneous item loadings. Test–retest reliability was assessed to evaluate the temporal stability of the I-BNPRQ. Missing data were inspected before analysis. Only questionnaires with sufficient item-level completeness for scale scoring and psychometric analysis were included. Participants who did not complete both administrations of the questionnaire were excluded from the test–retest reliability analysis only. A subsample of participants was invited to complete the questionnaire a second time after a two-week interval, which is considered adequate to minimise recall bias while ensuring stability of the construct being measured.

Only participants who completed both administrations were included in the analysis. Test–retest reliability was estimated using the intraclass correlation coefficient (ICC), calculated based on a two-way mixed-effects model with absolute agreement (ICC [1,3]). ICC values were interpreted according to established guidelines: values ≥ 0.75 indicate good reliability and ≥0.90 indicate excellent reliability. Group differences in continuous scale scores were examined using nonparametric tests (Mann–Whitney U and Kruskal–Wallis), given the data’s distributional characteristics. For analyses involving categorical outcomes, Generalised Linear Models (GLMs) were used where appropriate [32]. No additional analyses of convergent validity, discriminant validity, or measurement invariance were performed in the present study. These analyses were not part of the original validation plan and would have required additional external measures or larger, adequately powered subgroup samples.

Statistical significance was set at α = 0.05. All analyses were conducted using R version 4.3.3 (R Foundation for Statistical Computing, Vienna, Austria).

#### Sample Size Calculation

The initial sample size was determined using Watkins’ rule of thumb [28], requiring at least 10 observations per item. A post hoc power analysis assessed the adequacy of the sample (n = 196) for RMSEA analysis using the semPower() function from the *semPower* package in R. For an effect size of 0.08 (threshold for poor fit) with n = 196, 52 degrees of freedom, and a 5% significance level, the statistical power was 99.7%. The estimated sample size for 80% power under these conditions was n = 94.

### 2.4. Ethical Consideration

The study protocol was reviewed and approved by the local Institutional Ethics Committee (Palermo 1, Italy, protocol number BNPRQ 00 Vrs 1 n° 23/2024).

## 3. Results

A total of 196 nurses were included in the analysis. The sample was mostly female (n = 163, 82.7%), with a mean age of 42.06 years (SD 11.09), a mean professional experience of 17.57 years (SD 11.22), and a mean duration in the current position of 8.57 years (SD 8.53). Further sample characteristics are summarised in Table 1.

### 3.1. Content Validity

Twelve experts evaluated the relevance of each item. All items reached acceptable CVR values, ranging from 0.667 to 1.000, supporting their relevance at the content validity stage. The highest CVR value of 1.000 was obtained for several items, including lack of leadership support, lack of experienced nursing research mentors, lack of resources to facilitate nursing research, lack of research training opportunities, lack of incentive or reward for nurses to do research, lack of research knowledge or skills, lack of ideas for research project topics, and research relevance to nursing practice. Items with CVR values of 0.667 were retained after expert discussion because they were considered conceptually relevant to the construct and applicable to the Italian nursing context. Qualitative feedback indicated that the I-BNPRQ items were generally clear and relevant, and no additional items were introduced. Full CVR values are reported in Supplementary File S1. Qualitative feedback indicated that the I-BNPRQ items were clear and relevant, and no further modifications were required.

### 3.2. Structural Validity

Preliminary analyses indicated that the data were suitable for factor analysis. The overall KMO = 0.750 was optimal, and Bartlett’s test of sphericity was significant (χ^2^_(91)_ = 711.682, *p* < 0.001).

The original two-factor structure was first examined using CFA and showed poor fit (χ^2^_(64)_ = 215.438 (*p* < 0.001), RMSEA = 0.103 (90% CI: 0.081–0.125), CFI = 0.812, TLI = 0.793, SRMR = 0.078. Inspection of factor loadings identified problematic items (Items 1, 7, and 9; all λ < 0.40). The dimensionality of the Italian version was therefore further investigated using ESEM.

An initial two-factor ESEM solution showed partial improvement but remained suboptimal in terms of global fit: χ^2^_(52)_ = 152.374, *p* < 0.001; RMSEA = 0.089 (90% CI: 0.064–0.095); CFI = 0.872; TLI = 0.860; SRMR = 0.065.

The final ESEM yielded further improved fit indices: χ^2^_(52)_ = 118.963 (*p* < 0.0001), RMSEA = 0.074 (90% CI: 0.053, 0.080; *p* < 0.001), CFI = 0.906, TLI = 0.901, and SRMR = 0.048, supporting a two-factor structure. Factor 1, labelled “Research Resources”, comprised seven items and accounted for 35.37% of the variance. Factor 2, labelled “Personal Relevance of Research”, comprised four indicators and accounted for 25.14% of the variance. Items 7 and 9 were removed from the final latent structure because they showed low factor loadings and limited contribution to the Italian model. This may reflect the fact that, in the Italian context, the statements “Nursing research is not part of my job” and “Research is not relevant to nursing practice” may be interpreted less as measurable barriers and more as broader professional or cultural beliefs. Items 2 and 6 were merged because both addressed a closely related perceived lack of research competence, combining cognitive aspects of limited knowledge or skills with emotional aspects of intimidation or fear of research. This decision was based on both empirical overlap and conceptual coherence, and was discussed with reference to the original BNPRQ framework. Standardised factor loadings for the final model are presented in Table 2.

### 3.3. Reliability

Omega values were acceptable for both factors: ω = 0.87 for Research Resources and ω = 0.79 for Personal Relevance of Research. Test–retest reliability was adequate, with ICC = 0.79, 95% CI: 0.66–0.93, *p* < 0.001, indicating good temporal stability over the two-week interval.

### 3.4. Item Descriptive Statistics

The most frequently perceived barrier was “Lack of incentive/reward for nurses to do research” (Item 5), while the least perceived was “Research is not very interesting or valuable to me” (Item 14). The mean satisfaction score for research opportunities was M = 2.22 (1.11), and for personal participation, M = 2.78 (1.36) (Table 3).

### 3.5. Associations Between Sample Characteristics and Perceived Barriers

#### 3.5.1. Associations with Socio-Demographic and Professional Characteristics

Higher Research Resources scores were associated with shorter nursing career duration, fewer years at the current institution, younger age, and lower perceived research opportunities, whereas satisfaction was positively associated with this factor (ρs = −0.255, *p* < 0.001; ρs = −0.213, *p* < 0.01; ρs = −0.231, *p* < 0.01; ρs = −0.384, *p* < 0.001; ρs = 0.362, *p* < 0.001, respectively). For Personal Relevance of Research, nurses without prior experience in conducting a study reported higher barriers than those with such experience (Ws = 5259.5 and 5593.5; *p* = 0.016 and 0.043, respectively). This dimension was also positively associated with perceived research opportunities (ρs = 0.285, *p* < 0.001). Although Item 1 (“Lack of time to do research”) did not load on either factor, it showed the highest mean score and was perceived as a significant barrier by both staff nurses and clinical research nurses (χ^2^_(2)_ = 36.69, *p* < 0.001; χ^2^_(2)_ = 32.44, *p* < 0.001, respectively).

#### 3.5.2. Associations with Research Training

Nurses with advanced training reported lower Personal Relevance of Research barriers than those without advanced training (W = 4300, *p* = 0.011), whereas no significant differences emerged for Research Resources (W = 2969.5, *p* = 0.159). Likewise, Research Resources did not differ according to attendance at research training sessions or training in research tools (W = 4326, *p* = 0.545; W = 4207, *p* = 0.247, respectively). By contrast, nurses who had not attended research training sessions and those without training in research tools reported significantly higher Personal Relevance of Research barriers than their trained counterparts (W = 5814.5, *p* = 0.001; W = 6734, *p* < 0.001, respectively).

## 4. Discussion

This study aimed to cross-culturally adapt the BNPRQ into Italian and to evaluate its psychometric properties in a sample of Italian nurses. The findings provide preliminary evidence supporting the use of the I-BNPRQ to assess barriers to nurses’ participation in research within the Italian cultural and organisational context. The adaptation process was particularly relevant because research engagement is shaped not only by individual knowledge and motivation, but also by institutional infrastructure, leadership support, professional recognition, and the extent to which nursing research is embedded within healthcare organisations. The adaptation followed rigorous translation and cultural adaptation guidelines [25,28,29,30], ensuring relevance to the Italian context. This is crucial, as nurses’ research participation is influenced by country-specific factors such as healthcare infrastructure, education, and cultural attitudes toward research. The psychometric evaluation supported a two-factor structure, labelled Research Resources and Personal Relevance of Research. This structure was broadly consistent with the conceptual framework of the original BNPRQ, although the Italian version required item-level refinements. Therefore, the final I-BNPRQ should be considered a culturally adapted and psychometrically refined version of the BNPRQ rather than a direct linguistic validation of the original instrument. This distinction is important because item removal and item merging may affect direct comparability with studies using the original 15-item version. [24]. The removal of Items 7 and 9 may indicate that, in this sample, the perceived non-relevance of research to nursing practice and the belief that nursing research is not part of the professional role did not function as stable latent indicators. Rather than suggesting that these issues are unimportant, this result may reflect the changing professional discourse around evidence-based nursing in Italy, where research may be increasingly recognised as relevant in principle, while practical, organisational, and competency-related barriers continue to limit actual participation. The merging of Items 2 and 6 was also methodologically relevant. Both items concerned perceived inadequacy in relation to research, with one emphasising lack of knowledge or skills and the other emphasising intimidation or fear. In the Italian adaptation, these elements appeared to represent a closely related perceived barrier, here interpreted as a research confidence and skills deficit. This decision was supported by conceptual overlap, empirical behaviour, and discussion within the research team, including the involvement of one of the original BNPRQ developers. Interestingly, “lack of time” did not align with the Research Resources factor as in the original BNPRQ. This discrepancy may stem from nurses with greater access to research resources, such as Clinical Research Nurses and nurse managers, often facing heavier workloads and increased responsibilities [20,33,34], which further limit their research time [24]. Therefore, time constraints are more closely linked to overall workload and professional duties rather than mere availability of research resources, in accordance with studies highlighting time-related obstacles frequently reported by nurses, such as insufficient time to implement new ideas or read research, consistently ranking among the top barriers [35].

In the present study, lack of time emerged as a pervasive barrier, even though it did not load clearly on either latent factor. This finding suggests that time constraints may operate as a cross-cutting organisational issue rather than as a dimension-specific barrier. Future studies should investigate whether this item behaves differently across institutional settings and professional roles [21,22].

These findings should also be interpreted within the broader framework of evidence-based nursing, knowledge translation, and implementation science. Nurses’ participation in research is not only a matter of individual motivation, but also a prerequisite for strengthening research culture, improving the uptake of evidence, and supporting clinical decision-making. Barriers such as limited mentorship, lack of infrastructure, insufficient training, and weak recognition of research roles may reduce the capacity of nurses to contribute to knowledge generation and to the implementation of evidence in practice. From an implementation perspective, the I-BNPRQ may therefore be useful not only as a measurement instrument, but also as an organisational diagnostic tool to identify modifiable barriers and guide capacity-building strategies.

Nurses usually engage more in medical research than in nursing research [36]. In our study, only 10% acted as principal investigators. Loke et al. [37] attribute this to nurses’ lack of formal training in research methodologies, leading them to gravitate toward medical research where they feel more competent and supported. Berthelsen and Hølge-Hazelton [38] noted that nurses’ educational experiences often deprioritize nursing research, shaping their attitudes and preferences for medical research opportunities. Organisational culture within healthcare settings is also crucial in research engagement, with physicians’ dominance overshadowing nursing research initiatives. Nurses often prioritise medical research due to its prestige and accessibility over nursing research, which is viewed as less critical in such environments [36]. Additional barriers, like insufficient time, lack of authority to implement research findings, and limited awareness of nursing research opportunities, exacerbate this issue [27,39]. Munyisia et al. [40] observed that nurses in many settings allocate most of their time to medical duties rather than nursing research. This preference for medical research is reinforced by the perception that it presents fewer barriers than nursing research [35].

These findings underscore the urgent need for systemic changes to promote a more balanced approach to nursing research. Addressing key barriers such as time constraints, lack of authority, and inadequate training while fostering stronger institutional support is essential for encouraging greater participation in research [1]. The low mean satisfaction scores regarding research opportunities and personal involvement reflect general dissatisfaction among Italian nurses, likely linked to these barriers, particularly the lack of time and incentives [22,41,42]. Additionally, many nurses feel that research is not adequately integrated into their professional roles. To create more meaningful and accessible research opportunities, it is crucial to implement institutional changes that provide tangible rewards, recognise research contributions, and better integrate research into daily nursing activities. By fostering an environment where nursing research is equally prioritised and supported, nurses will have more opportunities and resources to contribute meaningfully to advancing the discipline [43,44,45].

### 4.1. Implications for Clinical Practice

The I-BNPRQ may support healthcare organisations in identifying specific barriers that prevent nurses from engaging in research. At the organisational level, the instrument could be used to map perceived barriers across units, professional roles, and institutional settings, thereby informing local strategies for research capacity building. For example, high scores in the Research Resources dimension may indicate the need to develop nursing research infrastructure, access to methodological support, research councils, or institutional mentorship pathways. Structured mentorship programmes should ideally involve experienced nurse researchers, clinical research nurses, academic partners, and research methodologists. These programmes could support nurses in formulating research questions, understanding study design, accessing ethical and administrative procedures, and translating clinical problems into researchable topics. Protected research time could be organised through dedicated research hours, project-based secondments, integration of research objectives into professional development plans, or formal recognition of research activities within workload planning. For the Personal Relevance of Research dimension, interventions should prioritise research literacy, methodological confidence, evidence appraisal, protocol development, ethical principles, data collection, and dissemination skills. Institutions could also monitor nurses’ research engagement through periodic assessment of perceived barriers, participation in research training, involvement in studies, production of practice-based projects, and contribution to evidence-based practice initiatives. In this sense, the I-BNPRQ may function as both an assessment instrument and a practical tool for guiding organisational improvement.

### 4.2. Limitations

This study has several limitations that should be considered when interpreting the findings. First, the use of convenience sampling may limit the generalisability of the results to the wider Italian nursing population. In addition, recruitment through online data collection and professional networks may have introduced selection bias, as nurses with a greater interest in research, evidence-based practice, or professional development were more likely to participate. From a methodological perspective, all data were collected using self-reported measures; therefore, the possibility of response bias, including social desirability bias, cannot be excluded.

A further limitation concerns the cultural adaptation process. Although this process included translation, back-translation, review by the original instrument’s developers, assessment by an expert panel, and evaluation of content validity, no preliminary pilot test was conducted with a separate sample of target users prior to field administration. This may have limited the extent to which nurses’ interpretation and understanding of each item could be fully documented prior to psychometric validation. Future studies should incorporate formal cognitive interviewing procedures with target nurses before large-scale psychometric validation in order to further strengthen semantic and experiential equivalence. Furthermore, to ensure appropriate cultural adaptation to the Italian context, the final version of the I-BNPRQ required the removal and merging of certain items. Although these modifications were methodologically justified, they may reduce direct comparability with the original BNPRQ.

Finally, this study provided evidence of content validity, structural validity, internal consistency, and test–retest reliability; however, additional psychometric properties, such as convergent validity, discriminant validity, criterion validity, responsiveness, and measurement invariance, were not assessed. Future studies should therefore confirm and extend these findings using larger, more heterogeneous, and representative samples, and should include comparative analyses across professional subgroups, healthcare settings, and different geographical areas.

## 5. Conclusions

Validating the I-BNPRQ is a significant step in understanding Italian nurses’ challenges in engaging with research activities. This study highlighted key barriers, including a lack of research resources and a lack of personal relevance, underscoring the complexities of integrating research into nursing practice in the Italian context. Use of the I-BNPRQ offers valuable insights and paves the way for developing strategic interventions to mitigate these barriers. Findings consistently demonstrate that institutional support, research training, and recognition of research roles are critical for enhancing nursing research participation. Addressing perceived lack of time and incentives and fostering an environment that values nursing research are essential for creating a culture where evidence-based practice thrives. The BNPRQ’s applicability in the Italian context reaffirms its utility in diverse healthcare systems, providing a robust framework for evaluating and overcoming barriers to nursing research globally. Future research should focus on broader implementations of the I-BNPRQ across varied healthcare settings to ensure a comprehensive understanding of regional differences and the unique needs of different nursing communities. By strengthening infrastructure and support for nursing research, Italian healthcare institutions can empower nurses to make significant contributions to advancing healthcare knowledge and practice.

## Figures and Tables

**Table 1:** Sample socio-demographic characteristics.

Variables	n (%)
**Gender**	
Female	163 (82.74%)
Male	33 (16.75%)
**Do you have direct experience in research?**	
No	128 (65.33%)
Yes	68 (34.67%)
**Have you ever conducted a nursing study?**	
No	94 (47.96%)
Yes	102 (52.04%)
**Did the course of study you completed include a session dedicated to clinical research?**	
No	76 (38.78%)
Yes	120 (61.22%)
**Do you think that your university education provided you with the appropriate tools to conduct research?**	
No	115 (58.67%)
Yes	81 (41.33%)
**Education**	
Bachelors degree	66 (34.51%)
First-level master’s degree	82 (41.62%)
Second-level master’s degree	41 (20.81%)
Advanced course	1 (0.51%)
PhD	4 (2.03%)
**Setting**	
Hospital	64 (32.49%)
Scientific Institute for Research, Hospitalisation and Healthcare	61 (30.96%)
University Hospital	47 (23.86%)
Primary Care	16 (8.12%)

Note: PhD: Doctor of Philosophy.

**Table 2:** Factor loadings from the Exploratory Structural Equation Model.

Items	Research Resources (F1) λ (SE)	Personal Relevance of Research (F2) λ (SE)
Lack of availability of experienced nursing research mentors (Item 3)	**0.616 (0.088)**	0.120 (0.045)
Lack of institutional nursing research infrastructure (Item 4)	**0.814 (0.069)**	0.093 (0.040)
Lack of incentive/reward for nurses to do research (Item 5)	**0.544 (0.064)**	0.111 (0.042)
Lack of financial or other resources to facilitate nursing research (Item 11)	**0.512 (0.075)**	0.129 (0.048)
Lack of accessibility to a Nursing Research Council (Item 12)	**0.759 (0.105)**	0.144 (0.050)
Lack of leadership support (Item 13)	**0.564 (0.100)**	0.125 (0.047)
Lack of research training opportunities (Item 15)	**0.712 (0.094)**	0.135 (0.049)
Lack of research knowledge or skills (Item 2 *merged* 6)	0.312 (0.043)	**0.951 (0.083)**
My training and educational background do not qualify me to conduct research (Item 8)	0.120 (0.045)	**0.888 (0.074)**
I do not have ideas for research project topics (Item 10)	0.115 (0.044)	**0.628 (0.083)**
Research is not very interesting or valuable to me (Item 14)	0.095 (0.041)	**0.563 (0.094)**

Note. λ = fully standardised estimates; SE = standard error.

**Table 3:** Item-level descriptive statistics.

Items	Mean	SD
**Research resources (F1)**	**3.89**	**0.62**
Lack of availability of experienced nursing research mentors (Item 3)	3.81	1.00
Lack of institutional nursing research infrastructure (Item 4)	3.85	1.02
Lack of incentive/reward for nurses to do research (Item 5)	4.36	0.78
Lack of financial or other resources to facilitate nursing research (Item 11)	4.06	0.81
Lack of accessibility to a Nursing Research Council (Item 12)	3.88	0.93
Lack of leadership support (Item 13)	3.94	0.95
Lack of research training opportunities (Item 15)	3.35	1.06
**Personal relevance of research (F2)**	**2.24**	**0.72**
Lack of research knowledge or skills (Item 2 merged 6)	2.90	0.95
My training and educational background do not qualify me to conduct research (Item 8)	2.27	1.09
I do not have ideas for research project topics (Item 10)	2.21	1.02
Research is not very interesting or valuable to me (Item 14)	1.58	0.85

Note. M = Mean; SD = Standard Deviation.

## Data Availability

The data presented in this study are available on reasonable request from the corresponding author.

## Supplementary Materials

The following supporting information can be downloaded at: https://www.mdpi.com/article/10.3390/healthcare14121793/s1, Supplementary File S1: Content Validity Procedures.

## Abbreviations

The following abbreviations are used in this manuscript:

BNPRQ	Barriers to Nurses’ Participation in Research Questionnaire
CFA	Confirmatory Factor Analysis
CFI	Comparative Fit Index
CI	Confidence Interval
CVR	Content Validity Ratio
EFA	Exploratory Factor Analysis
ESEM	Exploratory Structural Equation Modelling
GLM	Generalised Linear Model
I-BNPRQ	Italian Barriers to Nurses’ Participation in Research Questionnaire
ICC	Intraclass Correlation Coefficient
IQR	Interquartile Range
IRCCS	Scientific Institute for Research, Hospitalisation and Healthcare
KMO	Kaiser–Meyer–Olkin
MLR	Maximum Likelihood Robust
PI	Principal Investigator
RMSEA	Root Mean Square Error of Approximation
SD	Standard Deviation
SRMR	Standardised Root Mean Square Residual
TLI	Tucker–Lewis Index
