# Supplementary material for: Cross-Cultural Adaptation and Psychometric Testing of the Italian Barriers to Nursing Research Participation (I-BNPRQ)
Source: Healthcare (Basel). 2026 Jun 22;14(12):1793. doi: 10.3390/healthcare14121793 (PMC13299612; doi:10.3390/healthcare14121793)
Supplement: Supplementary file 1 [file healthcare-14-01793-s001.zip › healthcare-4273445-File S1.pdf]

Table S1. Content Validity Ratio of the I-BNPRQ items

Items	'Essential'	'Useful'	'Not Essential'	CVR	Essential
Lack of leadership support	12	0	0	1.000	Essential
Lack of institutional nursing research infrastructure	11	1	0	0.833	
Lack of experienced nursing research mentors	12	0	0	1.000	Essential
Lack of accessibility to a Nursing Research Council	10	1	1	0.667	Essential
Lack of resources to facilitate nursing research	12	0	0	1.000	Essential
Lack of research training opportunities	12	0	0	1.000	Essential
Lack of incentive/reward for nurses to do research	12	0	0	1.000	Essential
Lack of research knowledge or skills	12	0	0	1.000	Essential
I do not have ideas for research project topics	12	0	0	1.000	Essential
Research is not very interesting or valuable to me	10	0	2	0.667	Essential
Research is not relevant to nursing practice	12	0	0	1.000	Essential
My training/educatio n do not qualify me to conduct research	11	1	0	0.833	Essential
I feel intimidated by research	11	1	0	0.833	Essential

Nursing research is not part of my job	10	0	2	0.667	Essential
Lack of time to do research	10	1	1	0.667	Essential

Note. CVR = Content Validity Ratio. CVR values were calculated according to Lawshe's method based on the ratings of 12 experts. All items reached acceptable CVR values and were retained at the content validity stage.
